# Case of *Pyuria* Successfully Treated

**Published:** 1785

**Authors:** 


### X. *Caſe of* Pyuria *ſucceſsfully treated. By* the ſame.

MRS. P. a middle-aged lady, had been troubled with pain in making water for ſeveral years, and her urine was often bloody. Her complaints were at firſt thought to be occaſioned by a ſtone in the bladder; but, after taking a variety of medicines without any benefit, ſhe was ſearched, and no ſtone could be felt.

In

In the year 1782 ſhe applied to an eminent phyſician of this town, her complaints being then a great deal worſe. At this time ſhe began to be troubled with the piles; her urine was bloody, and voided with great pain, eſpecially after uſing any exerciſe; and ſhe diſcharged daily with her urine ſeveral ounces of a matter which reſembled pus more than mucus, (though it had ſomewhat of the viſcidity of mucus) and I think anſwered to the deſcription given by Sauvages of *Pyuria**.

Different medicines were adminiſtered to her, and ſome of them at firſt with ſeeming advantage; but after a ſhort period they ceaſed to produce any good effect, till, at length, in January, 1783, I adviſed her to try an infuſion of malt prepared in the following manner:

> Take of malt finely powdered ſix table ſpoonfuls; infuſe it all night, near the fire, in three pints of boiling water, and in the morning pour off the infuſion clear for uſe.

I was induced to recommend this remedy from recollecting an inſtance which had been communicated to me ſeveral years before, of

* Noſolog. Method. claſs. ix. gen. 28.

its its good effects in a similar case, and in 1782 I had been informed that the patient remained in good health, and had had no return of the complaint.

Mrs. P. began with the above infusion on the 3d of February, 1783, and continued to take three pints of it daily till the beginning of May, when she began to diminish the doses, but did not entirely leave it off till several months afterwards. She never made bloody urine (except once, and that after a long walk) after she began the use of the infusion; the discharge of viscid matter diminished very fast, and her urine was quite clear before May, 1783.

She took no other medicine after she began with the infusion, but adhered strictly to a milk and vegetable diet. She remains in good health at present, and has had no relapse.

